# Separation of Monoclonal Antibody Aggregates Using an Analytical Ultrafiltration Technique

**DOI:** 10.3390/membranes16060207

**Published:** 2026-06-10

**Authors:** Raja Ghosh, Mrunal Ingawale, Yves Durocher

**Affiliations:** 1 Department of Chemical Engineering, McMaster University, 1280 Main Street West, Hamilton, ON L8S 4L7, Canada; ingawalm@mcmaster.ca; 2 National Research Council of Canada, Montreal, QC H4P 2R2, Canada; yves.durocher@cnrc-nrc.gc.ca

**Keywords:** analytical ultrafiltration, membrane, monoclonal antibody, aggregates, analysis

## Abstract

Size exclusion chromatography is the industry-standard method for measuring aggregate content in monoclonal antibody samples. In this paper, we present an orthogonal analytical technique based on ultrafiltration for detecting and quantifying monoclonal antibody aggregates. The sample to be analyzed was injected into the system in the ultrafiltration mode, and the monomeric monoclonal antibody molecules were detected in the form of a permeate peak. The system was then switched to backflow mode, and the aggregates were recovered and detected in the form of a retained species peak. The aggregate content in a given sample was quantified based on the relative peak areas, akin to that in liquid chromatography. Two monoclonal antibodies were tested in this study using the proposed analytical ultrafiltration technique. Size exclusion chromatography served as the control technique. The data obtained using the two techniques were found to be in good agreement. The advantages and limitations of the proposed analytical ultrafiltration technique are discussed.

## 1. Introduction

Monoclonal antibodies (mAbs) are widely used for a range of therapeutic and diagnostic applications [1,2,3,4]. These are large macromolecules with complex structures. While such structural complexity allows them to perform their respective functions in biology, this also makes them prone to denaturation [5,6], fragmentation [7,8], aggregation [9,10,11,12], and chemical modifications such as oxidation [13,14], deamidation [15,16], and glycation [17]. Aggregation, which is a particularly common problem with most mAbs, is highly undesirable. Generally, aggregated forms of mAbs lack therapeutic activity [18,19]. Also, the presence of mAb aggregates in formulations has been linked to problems such as toxicity and immunogenicity [20,21]. Therefore, sensitive and reliable methods for detecting aggregates are critically important for ensuring both safety and efficacy of mAb products.

Size exclusion chromatography (SEC) is widely considered the standard method for analyzing mAb aggregates [22,23,24,25,26]. While this technique is extensively used in the biologics industry, it has some limitations [27,28,29,30,31,32,33]. It has been widely reported that SEC underestimates the mAb aggregate content in samples being tested [27,28,29,30,31,32,33]. One of the primary reasons for this is the incomplete recovery of proteins during SEC analysis due to adsorption by non-specific electrostatic or hydrophobic interaction [27,32,34]. Such interactions can take place with the stationary phase material as well as with the surface of components of chromatography hardware [32,34,35]. Also, there is preferentially greater adsorption of mAb aggregates on chromatography components when compared to the mAb monomer [34,36]. In addition to incomplete recovery, such interactions could also result in a change in elution characteristics, peak tailing, and band broadening [34]. Different strategies such as the use of arginine and mild detergents in the mobile phase, high ionic strength buffers, and high concentration of counter ions have been proposed for reducing non-specific interactions [34,35]. The use of titanium chromatography hardware in place of stainless steel has been shown to improve mAb aggregate recovery during SEC [34]. Column passivation and pre-conditioning steps have also been shown to be useful in reducing mAb aggregate adsorption on new columns [36]. Another reason for incorrect or subjective estimation of aggregate content during SEC-based analysis is that the mAb dimer peak is not always totally resolved from the mAb monomer peak at the baseline [27,28,34]. Consequently, certain data-filtering and interpretation approaches are used for peak integration, which could potentially lead to user-induced or software-induced subjectivity in aggregate estimation.

Due to the above limitations, orthogonal techniques such as sedimentation velocity analytical ultracentrifugation (SV-AUC) and asymmetric field flow fractionation (AFFF) are frequently used along with SEC to obtain more complete information [12,27,28,29,37,38,39,40]. Some of these techniques actually give more reliable information about mAb aggregate content. For instance, the percentage of soluble aggregates measured by SEC was found to be significantly lower than that obtained by SV-AUC [27]. Also, SEC is not suitable for detecting insoluble mAb aggregates due to their retention in frits and column pre-filters. A wide range of orthogonal techniques have been used to detect and quantify soluble and insoluble mAb aggregates, each technique having its own merits and demerits [12,39]. Aggregates are more hydrophobic and could therefore be separated from monomeric mAb using hydrophobic interaction chromatography [41,42]. Aggregates could also be separated from the monomeric form of mAbs based on differences in degree of interaction with cation exchange and mixed-mode media [43,44]. However, with most such chromatographic techniques, the samples would first have to be diluted in the mobile phase before separation could be carried out, and this could potentially alter the composition of the sample. For instance, dimer dissociation during sample dilution for SEC analysis has been reported in the literature [27].

**Figure 1:**
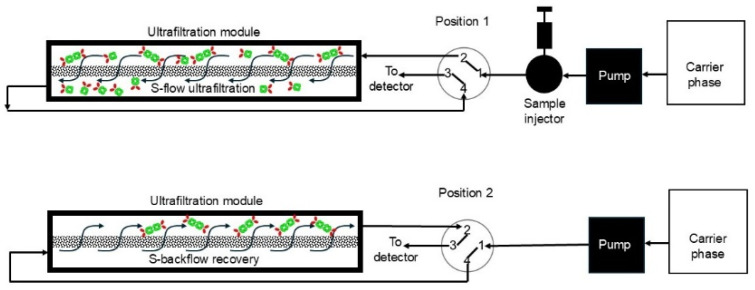
Experimental set-up and process schematic used for separation and analysis of monoclonal antibody aggregates using analytical ultrafiltration.

**Figure 2:**
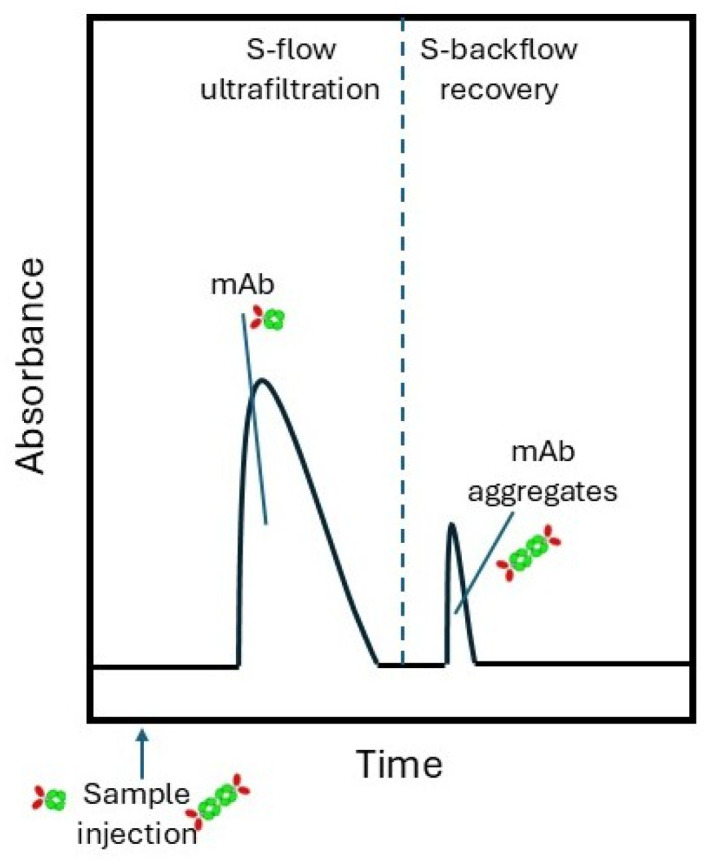
Idealized fractogram for the separation and analysis of monoclonal antibody aggregates using analytical ultrafiltration.

In this paper, we discuss an analytical ultrafiltration (AUF) technique for the separation, detection, and analysis of mAb aggregates. This method is based on a recently reported carrier phase ultrafiltration and backflow recovery (CPUFBR) technique that was developed for small- to medium-scale preparative protein purification [45]. The proposed AUF technique is a scaled-down version of the CPUFBR technique and has been further customized for an analytical application. The working hypothesis of the proposed AUF technique is summarized in Figure 1. An ultrafiltration module is fitted with a membrane that retains the mAb aggregates but allows the monomeric form of the mAb to permeate. The membrane module used for this technique is custom-designed for low dead-volume and allows the permeate to be collected in a uniform manner. This device is integrated with a liquid chromatography system using a four-port, two-position valve [45], as shown in Figure 1. The carrier phase from the liquid chromatography system is directed to port 1 of the valve, while the permeate from the membrane module is directed to port 4 of the valve. The liquid from port 3 of the valve is directed to the detectors of the liquid chromatography system. The sample to be analyzed is injected when the valve is in position 1. This results in the so-called S-flow ultrafiltration [45] pattern within the membrane module. The monomeric mAb molecules pass through the membrane and are detected in the form of a permeate (first) peak (see Figure 2). Once all the monomeric mAb has been removed in the permeate, the valve is switched to position 2 such that the species retained by the membrane is directed to port 2 of the valve, and from there, through port 3, to the detector. This results in the so-called S-backflow recovery [45] of the retained mAb aggregates, and these are detected in the form of a composite retained species (second) peak (see Figure 2).

The ultrafiltration membrane used for the proposed AUF technique discussed in this paper had a molecular weight cut-off (MWCO) of 100 kDa. Preliminary experiments were carried out using membranes having different MWCO ratings, and this particular membrane was found to be most suitable for this project. This membrane was selected based on the results of preliminary experiments, which showed that at the volumetric permeate flux (*J_v_*) used in the AUF experiments, monomeric mAb was freely transmitted through this membrane while aggregated mAb was retained. The passage of IgG class mAb (which has a molecular weight of ~150 kDa) through a 100 kDa MWCO-rated ultrafiltration membrane may seem counterintuitive. However, the MWCO rating is at best a rough guideline for membrane selection as retention is not only dependent on size but also on other factors such as operating pH, ionic strength, and the volumetric permeate flux [46,47,48]. There are reports in the literature where molecules significantly smaller than the membrane rating were substantially retained, while molecules larger than the membrane rating easily passed through [46,47,48]. Two mAbs were examined in this study. Trastuzumab (mAb1), in which aggregation was induced by repeated freeze–thawing, was used as the high aggregate-containing mAb sample. Another mAb (hIgG1-CD4) was used as the low aggregate-containing sample (mAb2). The aggregate content in these mAb samples was determined using the proposed AUF technique. The results obtained by AUF were compared with those obtained by SEC.

## 2. Materials and Methods

Polyethersulfone (PES) ultrafiltration membrane (OMEGA, part OT100 SHEET) with a molecular weight cut-off of 100 kDa was purchased from Pall Life Sciences (Mississauga, ON, Canada). Tricorn GL 10/300 column and Superdex 200 HR size exclusion chromatography media were purchased from GE Healthcare Bioscience (Uppsala, Sweden). A four-port, two-position valve (Cheminert) was purchased from VICI Valco (Houston, TX, USA). Sodium chloride (S7653), potassium chloride (P9541), sodium phosphate dibasic (S0876), and potassium phosphate monobasic (P5655) were purchased from Sigma-Aldrich (St. Louis, MO, USA). Trastuzumab (monoclonal antibody) was produced as described previously [49], while hIgG1-CD4 (monoclonal antibody, batch 12) was kindly donated by the Therapeutic Antibody Centre, Oxford University, United Kingdom. These monoclonal antibodies will be referred to in this paper as mAb1 and mAb2, respectively. Purified water (18.2 MΩ cm) used in preparing buffers and protein feed solutions was obtained from a Diamond™ NANOpure (Barnstead, Dubuque, IA, USA) water purification unit.

The monoclonal antibody samples were first tested using analytical SEC using a Tricorn GL 10/300 column packed with Superdex 200 HR media. Phosphate-buffered saline (PBS, pH 7.4) was used as the mobile phase, and the analyses were carried out at a flow rate of 0.1 mL/min. The volume of the mAb sample injected in the SEC experiments was 20 µL.

**Figure 3:**
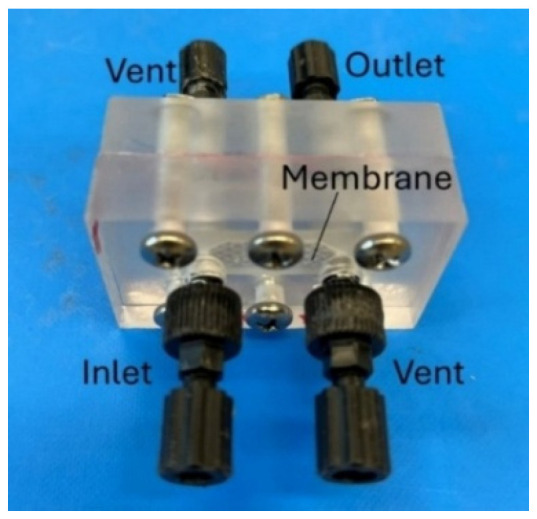
Membrane module used for analytical ultrafiltration.

The ultrafiltration membrane module (see Figure 3) used for conducting the AUF experiments discussed in this paper was designed and fabricated in-house. The effective area of the ultrafiltration membrane within the module was 1.33 cm^2^, the pressure limit being 1 MPa. The module, which was assembled by sandwiching the ultrafiltration membrane between a feed plate and a collection plate as discussed elsewhere [45], was integrated with an AKTA Prime Plus liquid chromatography system (GE Healthcare Biosciences, Montreal, QC, Canada) using appropriate tubing and a four-way, two-position valve. The AUF fractograms were obtained based on ultraviolet absorbance measurement at a 280 nm wavelength. The AUF technique consisted of two steps: ultrafiltration and backflow recovery (see Figure 1), both of which were carried out at a flow rate of 0.5 mL/min. During the ultrafiltration step, the permeate from the membrane module was directed to the UV detector of the liquid chromatography system, while during the backflow recovery step, the material retained by the ultrafiltration membrane was directed to the UV detector. Therefore, the output from the AUF technique for mAb aggregate analysis consisted of a permeate peak due to the mAb monomer and a retained material peak due to mAb aggregates (as represented in Figure 2). Phosphate-buffered saline (PBS, pH 7.4) was used as the carrier phase in the AUF experiments. This buffer was selected to ensure that the transmission of the monomeric form of the mAb through the ultrafiltration membrane was high. Earlier studies have shown that antibody transmission was higher in high-ionic-strength carrier phases with pH close to neutral [48]. The monoclonal antibody sample was injected using an appropriate sample injector with the AUF set-up in the ultrafiltration mode, this being the ultrafiltration step. Once the permeable species (the monomeric form of the mAb) present in the sample was completely removed in the permeate stream, the backflow recovery step was initiated by switching the position of the four-port, two-position valve.

## 3. Results and Discussion

**Figure 4:**
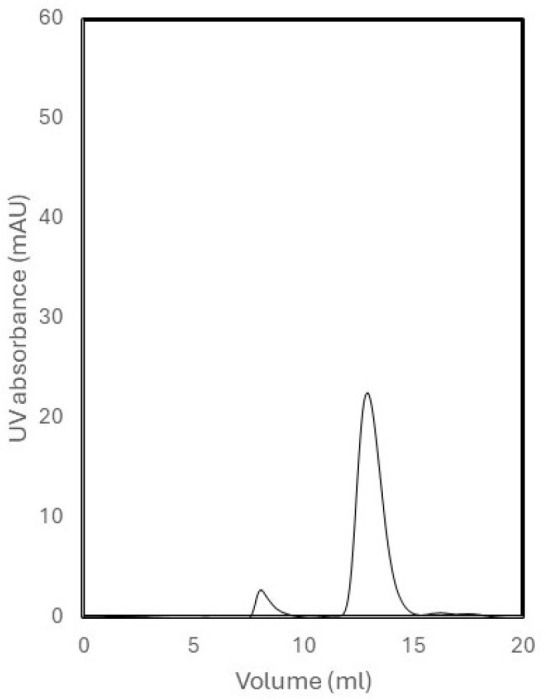
Analytical SEC chromatogram obtained with mAb1 monoclonal antibody (column: Tricorn GL 10/300, media: Superdex 200 HR, flow rate: 0.1 mL/min, sample volume: 20 µL, mAb conc.: 1 mg/mL).

**Figure 5:**
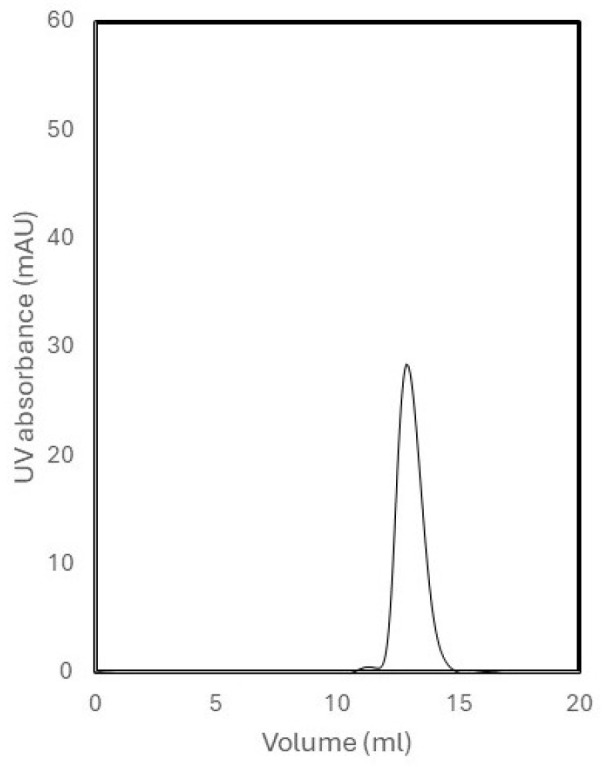
Analytical SEC chromatogram obtained with mAb2 monoclonal antibody (column: Tricorn GL 10/300, media: Superdex 200 HR, flow rate: 0.1 mL/min, sample volume: 20 µL, mAb conc.: 1 mg/mL).

The two monoclonal antibody samples were first analyzed using analytical size exclusion chromatography. These SEC experiments served as the control experiments for the proposed AUF technique. Figure 4 shows the SEC chromatogram obtained with mAb1, while Figure 5 shows the SEC chromatogram obtained with mAb2. The volume of the monoclonal antibody solution injected in these experiments was 20 µL, and the concentration was 1 mg/mL. The peaks were identified on the basis of molecular weight calibration. The peak integration data obtained from these chromatograms is summarized in Table 1. The aggregate content in mAb1 was 8.60%, while that in mAb2 was 2.05%. Based on the retention time of the aggregate peaks in the two chromatograms, it may be inferred that the aggregates present in the mAb1 sample were higher-order aggregates (e.g., trimers, tetramers), which were obtained in the void volume fraction of the SEC column. On the other hand, the aggregates present in mAb2 were predominantly dimers.

**Figure 6:**
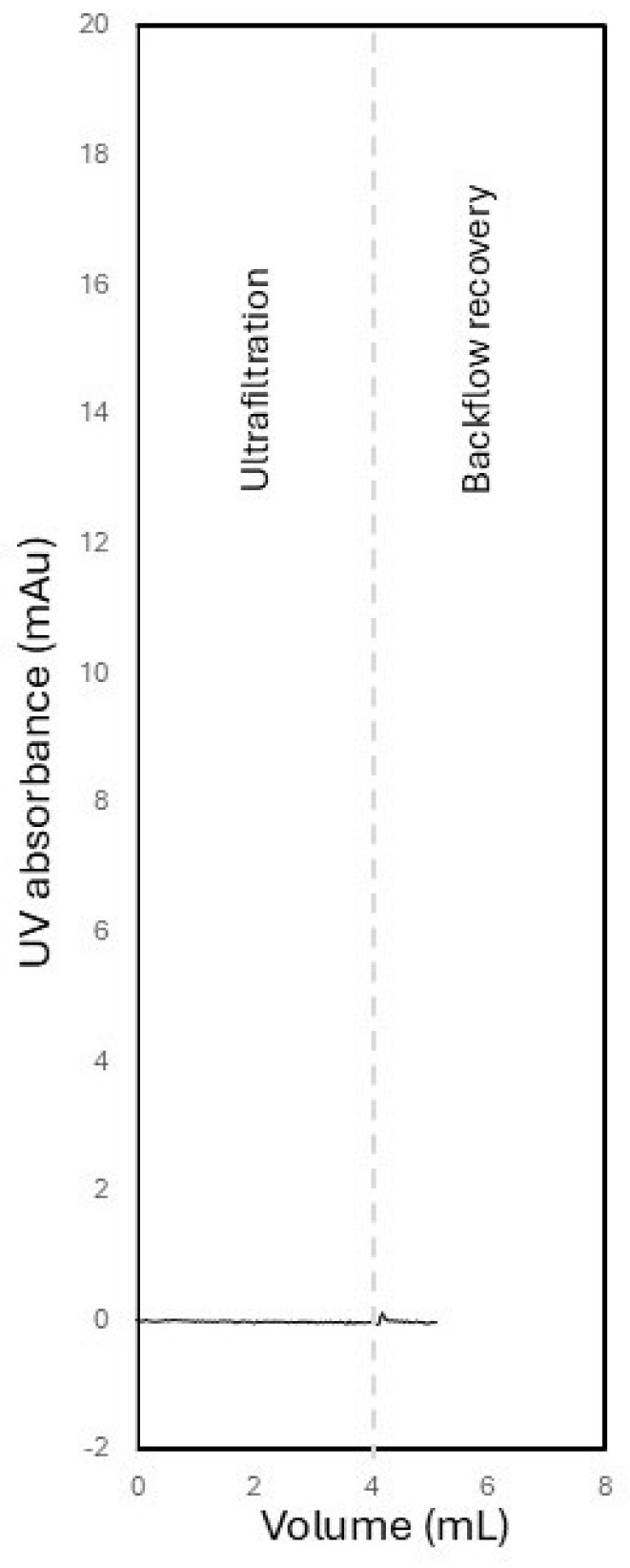
Analytical ultrafiltration fractogram obtained with “blank” sample (membrane: PES 100 kDa; flow rate: 0.5 mL/min; backflow: at 4 mL; sample volume: 10 µL).

The AUF experiment was first carried out using a “blank” sample, i.e., by injecting 10 µL of PBS (pH 7.4). The ultrafiltration and backflow recovery steps were carried out at the same flow rate, i.e., 0.5 mL/min, with backflow being initiated at 4 mL. The volumetric permeate flux in the ultrafiltration step of the experiment was therefore 6.27 × 10^−5^ m/s. This blank experiment was carried out to ascertain the extent to which the results obtained using AUF could be affected by experimental artifacts. Figure 6 shows the fractogram obtained from a typical blank experiment. As can be observed, there were no UV absorbance disturbances or drifts in the ultrafiltration phase of the experiment. There was a very slight disturbance in the UV signal (in the fractogram) when the flow was reversed using the four-port, two-position valve for the backflow recovery phase of the experiment. However, this disturbance manifested itself in the form of a minuscule peak with a very small peak area. It was therefore unlikely to impact the results obtained in the mAb aggregate analysis experiments (as discussed later in the paper based on comparison of peak area data).

**Figure 7:**
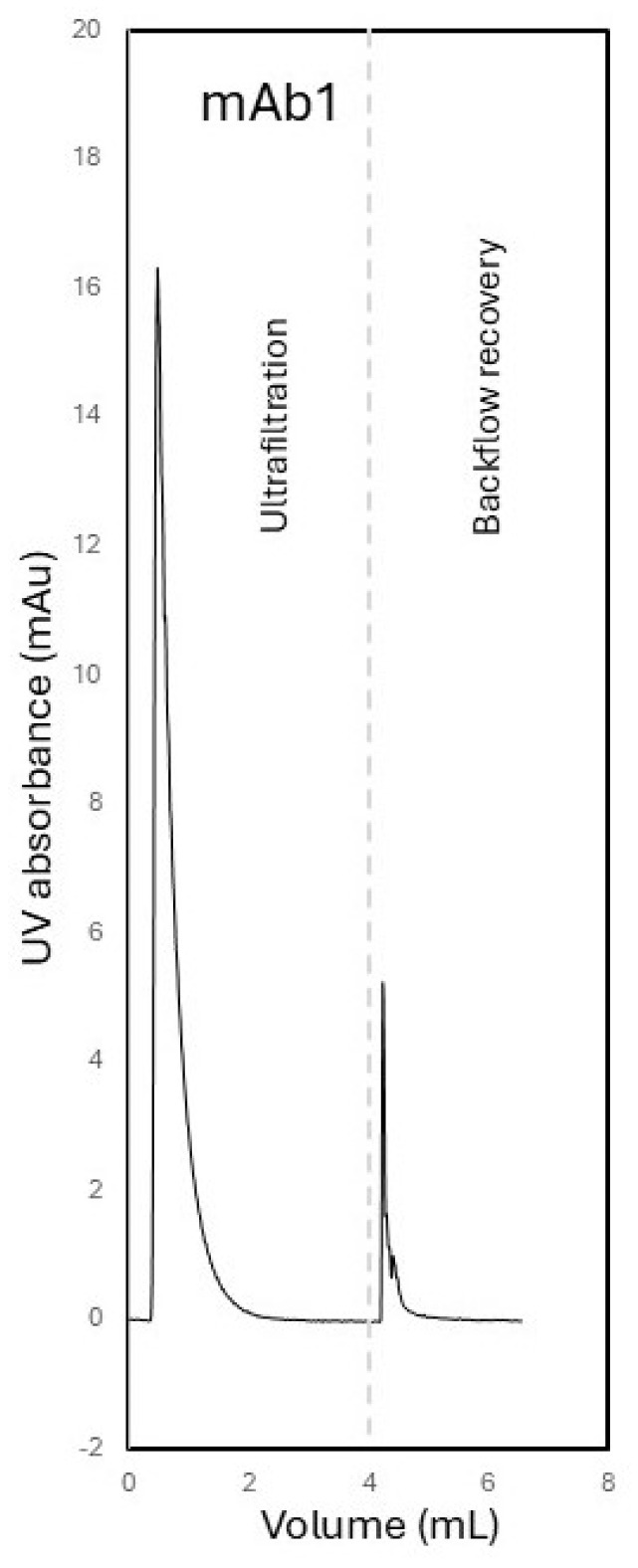
Analytical ultrafiltration fractogram obtained with mAb1 monoclonal antibody sample (membrane: PES 100 kDa; flow rate: 0.5 mL/min; backflow: at 4 mL; sample volume: 10 µL, mAb conc.: 1 mg/mL).

Figure 7 shows the fractogram obtained during the analysis of mAb1 using AUF. The volume of monoclonal antibody solution injected in these experiments was 10 µL, and the concentration was 1 mg/mL. In this experiment, which was also carried out using a flow rate of 0.5 mL/min, two distinct peaks were obtained, one corresponding to the permeable species (i.e., the mAb monomer) and the other corresponding to the retained species (i.e., the mAb aggregates). The backflow was initiated after 4 mL to allow the UV absorbance signal following the first peak to reach the baseline. This ensured that the permeable species was completely removed from the membrane module before the retained species were recovered. The aggregate content of mAb1 was determined by peak integration data obtained from five repeat experiments (see Table 2). The identification of the aggregates peak in an AUF fractogram was based on the expectation that aggregated species would be quantitatively retained by the 100 kDa MWCO membrane, as well as based on comparison with the corresponding SEC chromatogram. A comparison of data shown in Table 1 and Table 2 would suggest that the aggregate content data (for mAb1) obtained using the two analytical techniques (i.e., analytical SEC and AUF) were in good agreement.

**Figure 8:**
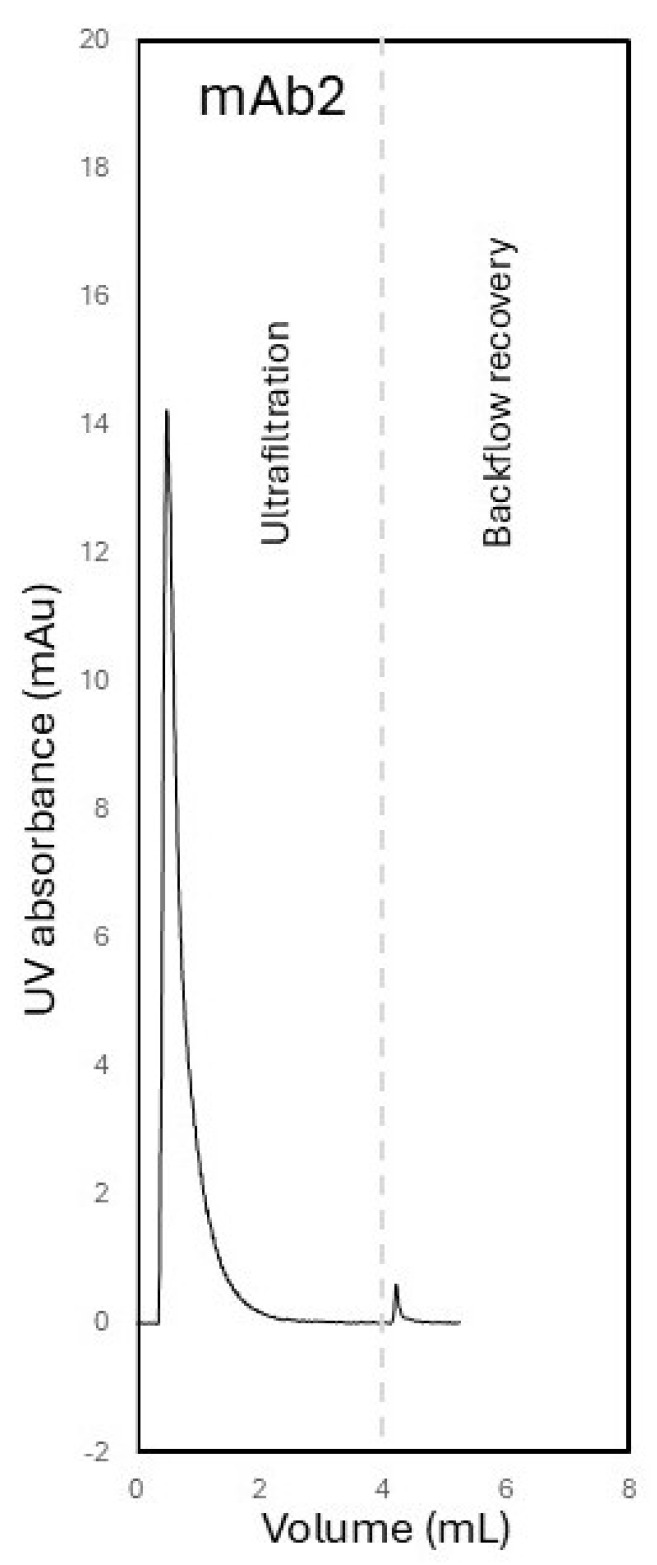
Analytical ultrafiltration fractogram obtained with mAb2 monoclonal antibody (membrane: PES 100 kDa; flow rate: 0.5 mL/min; backflow: at 4 mL; sample volume: 10 µL; mAb conc.: 1 mg/mL).

Figure 8 shows the fractogram obtained during the analysis of mAb2 using AUF. This experiment was carried out using an identical protocol to that used for mAb1, i.e., flow rate of 0.5 mL/min with backflow recovery initiated at 4 mL. The volume of monoclonal antibody solution injected in this experiment was 10 µL, and the concentration was 1 mg/mL. Consistent with expectation, the recovered species peak obtained with mAb2 was significantly smaller than that obtained with mAb1. The mAb2 analysis experiments were also carried out using five repeats and the average aggregate content was determined (see Table 2). The mAb2 aggregate content data obtained using the two analytical techniques (i.e., analytical SEC and AUF) were also found to be in good agreement.

The monoclonal antibody monomer and aggregate peaks obtained using the analytical ultrafiltration technique were well resolved and area under the curve calculation for peak area determination was quite straightforward. The baseline was very stable, and the area of each peak was determined by integration of the respective area above the baseline. Indeed, while using this technique, the resolution between the two peaks could be adjusted (i.e., increased or decreased) by changing the volume at which the backflow recovery mode is initiated. In size exclusion chromatography, the monoclonal antibody monomer and aggregate peaks are not always well resolved, leading to subjectivity in peak integration. The average area under the curve (based on five experiments) of the minuscule disturbance peaks obtained in the “blank” experiment (see Figure 6) was 0.009 mAu mL, while the corresponding averaged values for recovered species peaks obtained with mAb1 (see Figure 7) and mAb2 (see Figure 8) were 1.034 mAu mL and 0.223 mAu mL, respectively. Based on this, it may be safely inferred that the aggregate content data obtained for both mAb samples were not tainted by any artifact generated by disturbance during the changeover from the ultrafiltration step to the backflow recovery step.

Based on the results discussed above, it would be fair to say that the proposed AUF technique represents a promising new approach for measuring mAb aggregate content. The AUF techniques could be refined and developed further as an orthogonal technique with respect to SEC. The excellent agreement in aggregate content data (for the two mAbs tested in this study) obtained with SEC and AUF is encouraging and would indicate that AUF would potentially be widely applicable. The typical sample analysis run time for the AUF technique was about 10 min, which compared very favorably with some of the more advanced high-performance liquid chromatography (HPLC) techniques currently used in the industry [50]. With the AUF technique, the volume of carrier (or mobile) phase used per run was about 5 mL, which was also quite low compared to a typical HPLC run. These features make it particularly attractive for use in screening formulation buffers for mAb drug products. However, the main limitation of AUF is that all aggregated species are lumped together in the retained species peak. Therefore, the proposed technique, while being suitable for measuring the net mAb aggregate content, cannot be used for determining the composition of the different types of aggregates in a given sample, i.e., dimers, trimers, and so on. In some applications, such as the study of mechanisms involved in aggregate formation, it is important to know the relative content of the different aggregated species. On the other hand, when aggregates are split over multiple and sometimes unresolved peaks, the chance of integration errors and measurement subjectivity is very high. The fact that all aggregated species are consolidated in a single peak in the AUF technique would suggest that the net aggregate measurement data obtained using this technique would be more reliable. Also, the AUF technique measures both soluble and insoluble mAb aggregates. Therefore, AUF could potentially be used as an orthogonal analytical technique for routine mAb aggregate content measurement for batch clearance. Whether the approach used in this study could be used to further segregate different aggregated species—i.e., soluble aggregates from insoluble aggregates—will be examined in future studies. The effects of solution conditions on protein–membrane interaction, and its potential impact on the analytical ultrafiltration technique, will also be investigated.

## 4. Conclusions

The proposed analytical ultrafiltration technique was shown to be suitable for measuring aggregate content in monoclonal antibody samples. Blank experiments showed that the aggregate content data obtained using the proposed technique was not likely to be tainted by any artifact. The versatility of the technique was demonstrated by analyzing two different monoclonal antibody samples that had different levels of aggregate content. The results obtained using the proposed technique were compared with those obtained using size exclusion chromatography, which served as the control technique. The aggregate content measurements obtained using the two techniques were found to be in good agreement. The proposed analytical ultrafiltration technique could therefore be developed further to potentially serve as an orthogonal technique with respect to size exclusion chromatography for batch clearance of monoclonal antibody samples. The main limitation of the analytical ultrafiltration technique is that it measures all aggregates as one fraction. Therefore, it cannot be used for determining the composition of the different types of aggregates, such as dimers, trimers and so on, in a given sample. However, for the very same reason, the net aggregate content measurement obtained using the analytical ultrafiltration technique is likely to be more reliable than size exclusion chromatography as the different aggregate species are detected and measured as a composite peak and are not distributed and thereby diluted in multiple peaks.

## Figures and Tables

**Table 1:** Monoclonal antibody aggregate content as determined by analytical SEC.

Antibody	Percentage of Aggregates
mAb1	8.60
mAb2	2.05

**Table 2:** Monoclonal antibody aggregate content as determined by analytical ultrafiltration.

Antibody	Percentage of Aggregates
mAb1	8.89 (SD = 0.69)
mAb2	2.36 (SD = 0.11)

## Data Availability

The original contributions presented in this study are included in the article. Further inquiries can be directed to the corresponding author.

## References

[1] Adams G.P., Weiner L.M. (2005). Monoclonal antibody therapy of cancer. Nat. Biotechnol..

[2] Taylor P.C., Adams A.C., Hufford M.M., De La Torre I., Winthrop K., Gottlieb R.L. (2021). Neutralizing monoclonal antibodies for treatment of COVID-19. Nat. Rev. Immunol..

[3] Hale G., De Vos J., Davy A.D., Sandra K., Wilkinson I. (2024). Systematic analysis of Fc mutations designed to reduce binding to Fc-gamma receptors. mAbs.

[4] Durocher Y., Butler M. (2009). Expression systems for therapeutic glycoprotein production. Curr. Opin. Biotechnol..

[5] Schön A., Freire E. (2021). Reversibility and irreversibility in the temperature denaturation of monoclonal antibodies. Anal. Biochem..

[6] Lazar K.L., Patapoff T.W., Sharma V.K. (2010). Cold denaturation of monoclonal antibodies. mAbs.

[7] Gaza-Bulseco G., Liu H. (2008). Fragmentation of a recombinant monoclonal antibody at various pH. Pharm. Res..

[8] Salinas B.A., Sathish H.A., Shah A.U., Carpenter J.F., Randolph T.W. (2010). Buffer-dependent fragmentation of a humanized full-length monoclonal antibody. J. Pharm. Sci..

[9] Vázquez-Rey M., Lang D.A. (2011). Aggregates in monoclonal antibody manufacturing processes. Biotechnol. Bioeng..

[10] van der Kant R., Karow-Zwick A.R., Van Durme J., Blech M., Gallardo R., Seeliger D., Aßfalg K., Baatsen P., Compernolle G., Gils A. (2017). Prediction and reduction of the aggregation of monoclonal antibodies. J. Mol. Biol..

[11] Kuzman D., Bunc M., Ravnik M., Reiter F., Žagar L., Bončina M. (2021). Long-term stability predictions of therapeutic monoclonal antibodies in solution using Arrhenius-based kinetics. Sci. Rep..

[12] São Pedro M.N., Klijn M.E., Eppink M.H., Ottens M. (2022). Process analytical technique (PAT) miniaturization for monoclonal antibody aggregate detection in continuous downstream processing. J. Chem. Technol. Biotechnol..

[13] Gupta S., Jiskoot W., Schöneich C., Rathore A.S. (2022). Oxidation and deamidation of monoclonal antibody products: Potential impact on stability, biological activity, and efficacy. J. Pharm. Sci..

[14] Kaur H. (2021). Stability testing in monoclonal antibodies. Crit. Rev. Biotechnol..

[15] Yan Q., Huang M., Lewis M.J., Hu P. (2018). Structure based prediction of asparagine deamidation propensity in monoclonal antibodies. mAbs.

[16] Huang L., Lu J., Wroblewski V.J., Beals J.M., Riggin R.M. (2005). In vivo deamidation characterization of monoclonal antibody by LC/MS/MS. Anal. Chem..

[17] Wei B., Berning K., Quan C., Zhang Y.T. (2017). Glycation of antibodies: Modification, methods and potential effects on biological functions. mAbs.

[18] Sreenivasan S., Jiskoot W., Rathore A.S. (2021). Rapid aggregation of therapeutic monoclonal antibodies by bubbling induced air/liquid interfacial and agitation stress at different conditions. Eur. J. Pharm. Biopharm..

[19] Bansal R., Dash R., Rathore A.S. (2020). Impact of mAb aggregation on its biological activity: Rituximab as a case study. J. Pharm. Sci..

[20] Ahmadi M., Bryson C.J., Cloake E.A., Welch K., Filipe V., Romeijn S., Hawe A., Jiskoot W., Baker M.P., Fogg M.H. (2015). Small amounts of sub-visible aggregates enhance the immunogenic potential of monoclonal antibody therapeutics. Pharm. Res..

[21] Pham N.B., Meng W.S. (2020). Protein aggregation and immunogenicity of biotherapeutics. Int. J. Pharm..

[22] Woodard J., Lau H., Latypov R.F. (2013). Nondenaturing size-exclusion chromatography-mass spectrometry to measure stress-induced aggregation in a complex mixture of monoclonal antibodies. Anal. Chem..

[23] Goyon A., D’Atri V., Colas O., Fekete S., Beck A., Guillarme D. (2017). Characterization of 30 therapeutic antibodies and related products by size exclusion chromatography: Feasibility assessment for future mass spectrometry hyphenation. J. Chromatogr. B.

[24] Yan Y., Xing T., Huang X., Peng W., Wang S., Li N. (2024). Affinity-resolved size exclusion chromatography coupled to mass spectrometry: A novel tool to study the attribute-and-function relationship in therapeutic monoclonal antibodies. Anal. Chem..

[25] D’Atri V., Imiołek M., Quinn C., Finny A., Lauber M., Fekete S., Guillarme D. (2024). Size exclusion chromatography of biopharmaceutical products: From current practices for proteins to emerging trends for viral vectors, nucleic acids and lipid nanoparticles. J. Chromatogr. A.

[26] Fekete S., Beck A., Veuthey J.L., Guillarme D. (2014). Theory and practice of size exclusion chromatography for the analysis of protein aggregates. J. Pharm. Biomed. Anal..

[27] Gandhi A.V., Pothecary M.R., Bain D.L., Carpenter J.F. (2017). Some lessons learned from a comparison between sedimentation velocity analytical ultracentrifugation and size exclusion chromatography to characterize and quantify protein aggregates. J. Pharm. Sci..

[28] Gabrielson J.P., Brader M.L., Pekar A.H., Mathis K.B., Winter G., Carpenter J.F., Randolph T.W. (2007). Quantitation of aggregate levels in a recombinant humanized monoclonal antibody formulation by size-exclusion chromatography, asymmetrical flow field flow fractionation, and sedimentation velocity. J. Pharm. Sci..

[29] Carpenter J.F., Randolph T.W., Jiskoot W., Crommelin D.J., Middaugh C.R., Winter G. (2010). Potential inaccurate quantitation and sizing of protein aggregates by size exclusion chromatography: Essential need to use orthogonal methods to assure the quality of therapeutic protein products. J. Pharm. Sci..

[30] Goyon A., Fekete S., Beck A., Veuthey J.L., Guillarme D. (2018). Unraveling the mysteries of modern size exclusion chromatography-the way to achieve confident characterization of therapeutic proteins. J. Chromatogr. B.

[31] Gjoka X., Schofield M., Cvetkovic A., Gantier R. (2014). Combined Protein A and size exclusion high performance liquid chromatography for the single-step measurement of mAb, aggregates and host cell proteins. J. Chromatogr. B.

[32] Fekete S., Kizekai L., Saison Y.T., Lawrence N., Shiner S., Lauber M. (2022). Investigating the secondary interactions of packing materials for size-exclusion chromatography of therapeutic proteins. J. Chromatogr. A.

[33] Goyon A., Sciascera L., Clarke A., Guillarme D., Pell R. (2018). Extending the limits of size exclusion chromatography: Simultaneous separation of free payloads and related species from antibody drug conjugates and their aggregates. J. Chromatogr. A.

[34] Murisier A., Andrie M., Fekete S., Lauber M., D’Atri V., Iwan K., Guillarme D. (2022). Direct coupling of size exclusion chromatography and mass spectrometry for the characterization of complex monoclonal antibody products. J. Sep. Sci..

[35] Ejima D., Yumioka R., Arakawa T., Tsumoto K. (2005). Arginine as an effective additive in gel permeation chromatography. J. Chromatogr. A.

[36] Yumioka R., Sato H., Tomizawa H., Yamasaki Y., Ejima D. (2010). Mobile phase containing arginine provides more reliable SEC condition for aggregation analysis. J. Pharm. Sci..

[37] Demeule B., Shire S.J., Liu J. (2009). A therapeutic antibody and its antigen form different complexes in serum than in phosphate-buffered saline: A study by analytical ultracentrifugation. Anal. Biochem..

[38] Bou-Assaf G.M., Budyak I.L., Brenowitz M., Day E.S., Hayes D., Hill J., Majumdar R., Ringhieri P., Schuck P., Lin J.C. (2022). Best practices for aggregate quantitation of antibody therapeutics by sedimentation velocity analytical ultracentrifugation. J. Pharm. Sci..

[39] Le Basle Y., Chennell P., Tokhadze N., Astier A., Sautou V. (2020). Physicochemical stability of monoclonal antibodies: A review. J. Pharm. Sci..

[40] Larson N.R., Bou-Assaf G.M. (2023). Increasing the Resolution of Field-Flow Fractionation with Increasing Crossflow Gradients. Anal. Chem..

[41] Wang L., Hale G., Ghosh R. (2006). Non-size-based membrane chromatographic separation and analysis of monoclonal antibody aggregates. Anal. Chem..

[42] Madadkar P., Umatheva U., Hale G., Durocher Y., Ghosh R. (2017). Ultrafast separation and analysis of monoclonal antibody aggregates using membrane chromatography. Anal. Chem..

[43] Chen S., Lau H., Brodsky Y., Kleemann G.R., Latypov R.F. (2010). The use of native cation-exchange chromatography to study aggregation and phase separation of monoclonal antibodies. Protein Sci..

[44] Gao D., Wang L.L., Lin D.Q., Yao S.J. (2013). Evaluating antibody monomer separation from associated aggregates using mixed-mode chromatography. J. Chromatogr. A.

[45] Ghosh R. (2024). A Carrier Phase Ultrafiltration and Backflow Recovery Technique for Purification of Biological Macromolecules. Membranes.

[46] Saksena S., Zydney A.L. (1994). Effect of solution pH and ionic strength on the separation of albumin from immunoglobulins (IgG) by selective filtration. Biotechnol. Bioeng..

[47] Ghosh R. (2001). Fractionation of biological macromolecules using carrier phase ultrafiltration. Biotechnol. Bioeng..

[48] Ghosh R., Wan Y., Cui Z., Hale G. (2003). Parameter scanning ultrafiltration: Rapid optimisation of protein separation. Biotechnol. Bioeng..

[49] Raymond C., Robotham A., Spearman M., Butler M., Kelly J., Durocher Y. (2015). Production of α2, 6-sialylated IgG1 in CHO cells. mAbs.

[50] Cernosek T., Jain N., Dalphin M., Behrens S., Wunderli P. (2024). Accelerated development of a SEC-HPLC procedure for purity analysis of monoclonal antibodies using design of experiments. J. Chromatogr. B.

